# Low- and High-Energy Falls With Associated Traumatic Brain Injury: Epidemiology and Outcomes

**DOI:** 10.7759/cureus.80115

**Published:** 2025-03-05

**Authors:** Shameeke V Taylor, Tirth Patel, Connor Welsh

**Affiliations:** 1 Emergency Medicine, Icahn School of Medicine at Mount Sinai, New York City, USA; 2 Emergency Medicine, Mount Sinai Morningside, New York City, USA

**Keywords:** closed head injury, fall injury, high-energy trauma, low-energy trauma, traumatic brain injury

## Abstract

Introduction: Falls are among the most common causes of injuries treated in emergency departments (ED), accounting for approximately 30% of all injury-related ED visits. Falls are also a significant cause of traumatic brain injury (TBI), a fact for individuals across the aging spectrum. Despite an abundance of information on the association between falls and TBI, limited investigation has occurred into the differences in population characteristics and outcomes based on fall energy (low-energy vs. high-energy falls) for individuals with TBI due to unintentional falls.

Methods: We conducted a retrospective analysis of patients with diagnosed TBI at an urban trauma center. Hospital data were obtained from the Department of Surgery’s trauma registry, a subset of the National Trauma Registry of the American College of Surgeons, for the years 2017-2022. Low-energy falls were defined as a fall from ground level to three feet in height, and high-energy falls were defined as falls from greater than three feet. Individuals with TBI due to low and high-energy falls were compared using descriptive, univariate, and multivariate methods. Fall height was also used as a continuous variable to examine bivariate relationships with clinical characteristics in this cohort.

Results: This study identified 760 low-energy falls and 93 high-energy falls with associated TBI. Approximately 60% of the cohort was male, 14.5% identified as Black race, 20.1% utilized Medicaid, 12.2% had moderate-severe TBI, 15.6% had orthopedic injuries, and 7.3% died during hospitalization. Older age and Medicare insurance were associated with low energy falls. Younger age, orthopedic injuries, and alcohol use at the time of injury were associated with high-energy falls. No association was found between energy level and sex, length of stay, injury severity score (ISS), Glasgow coma scale (GCS) score on arrival, Medicaid usage, complication burden, TBI severity, disposition type, and death. Increasing fall height was associated with age, lower GCS score, greater ISS, and ventilator days. Multivariate analysis found that age and alcohol use were predictive of fall energy.

Conclusion: Overall, patients with high-energy falls with associated TBI were younger and were more likely to develop fractures and utilize alcohol at the time of injury. High-energy falls did not lead to more severe injuries as demonstrated by nonsignificant differences in ISS, GCS, TBI severity, and death. Targeted prevention programs should be designed to reduce traumatic injury. Further study is needed to evaluate long-term patient outcomes in this population.

## Introduction

Falls are among the most common causes of injuries treated in emergency departments (ED), accounting for approximately 30% of all injury-related ED visits [1]. The annual cost of treatment for fall-related injuries in older adults is thought to exceed $50 billion alone without accounting for falls in younger individuals [2]. This number will likely increase over time with the growth of the older population in the United States, as older adults are living longer and healthier lives with current medical knowledge and technological advances [3]. Falls are a significant cause of traumatic brain injury (TBI), a fact for individuals across the aging spectrum. In children ages 0 to 17, falls are the cause of almost half (49%) of TBI-related ED visits. Similarly, for middle-aged and older adults, unintentional falls are the leading cause of TBI-related ED visits and hospitalizations, accounting for 81% of TBI-related ED visits for older adults [4,5]. Despite an abundance of information on the association between falls and TBI, limited investigation has occurred into the differences in population characteristics and outcomes based on fall energy for individuals with TBI due to unintentional falls.

Falls are typically differentiated based on the potential energy involved, which is associated with the height of the fall to impact with a surface. High-energy falls refer to falls associated with significant energy transfer, which is predicated by increasing height leading to higher energy. Low-energy falls are typically falls of lower height, but what constitutes low energy varies in the literature, from ground level to one meter [6] and up to three meters [7] depending upon the study. Forty percent of injuries that result in TBI are due to low-energy falls [8]. Low-energy falls are the predominant trauma mechanism for older adults, leading to injury severities similar to high-energy mechanisms in younger patients [9]. However, the number of high-energy geriatric falls is increasing over time and is associated with worsened levels of injury and mortality [10]. Individuals recovering from head injuries often experience cognitive and neuromotor impairments [11]. These effects can include memory loss, dizziness, and problems with gait and balance, which increase the risk of additional falls and head injuries [11]. Further increasing their risk of falls and subsequent TBI are factors such as seizures, fatigue, and substance abuse that are associated with the initial head injury. The physical and neurocognitive sequelae after TBI have been linked to the severity of injury [12], with more severe injuries often resulting from direct head impacts and increasing fall height or energy [13]. Data on energy levels regarding falls, risk factors, and outcomes have either focused on one type (low or high energy mechanism) [10, 14], focused on rural populations [7], and/or featured populations outside of the United States [15]. Further evaluation is needed to better understand the epidemiological differences in patients with resulting TBI due to high- and low-energy falls and associated risk factors in a U.S. urban at-risk population.

Current information on risk factors for low- and high-energy fall-induced TBI in a US urban population is limited. However, identifying risk factors for serious TBI is an important initial step in developing effective TBI prevention strategies. This study aims to evaluate factors associated with fall-induced TBI in low- and high-energy falls and characterize the differences in demographic and clinical presentations between these two groups.

## Materials and methods

This was a retrospective analysis of trauma patients who were entered into the trauma registry of Mount Sinai Morningside Hospital, New York City, NY, USA. Data for this registry were previously collected as part of the National Trauma Data Bank, the largest available registry of trauma data, which is managed by the American College of Surgeons. Institutional informed consent was not required for this retrospective study of de-identified data based on a review by the Internal Review Board of Mount Sinai Hospital.

We designed a retrospective cohort study of patients with TBI identified in the trauma registry. The cohort included patients of all age groups who sustained a TBI due to unintentional falls and were treated at our level 2 academic trauma center and tertiary referral center between January 1, 2017, and August 30, 2022. Patients who were treated primarily at our trauma center or transferred from outside hospitals in our health system for further management were included. Traumatic brain injury was identified with Abbreviated Injury Scale (AIS) codes in the registry. 

We examined differences in patient characteristics and outcomes after TBI due to low-energy versus high-energy falls. Low-energy falls were defined as a fall from ground level to three feet in height. High-energy falls were defined as falls greater than three feet in height. These definitions are consistent with prior literature on the subject that delineates one meter, or approximately three feet, as the upper limit threshold of height for which low-energy falls are considered [6, 16, 17]. Fall height was also used as a variable to examine bivariate relationships with clinical characteristics in this cohort.

The patient characteristics examined included age, sex, race/ethnicity, TBI severity, Glasgow coma scale (GCS) score, injury severity score (ISS), alcohol use, presence of orthopedic injuries, complication burden, insurance type, and hospital disposition. We also examined the duration of mechanical ventilation, intensive care unit (ICU), and total hospital stay. The severity of TBI was classified as mild, moderate, or severe based on the GCS score ranges of 13 to 15, 9 to 12, and three to eight, respectively. These GCS scores were documented on patient arrival at the emergency department. The presence of orthopedic injuries was determined based on the note of orthopedic surgeon consultation and evidence of a traumatic fracture based on the AIS diagnosis code. Ventilator use was used as a surrogate marker for intubation during the hospitalization. Alcohol use near the time of injury was determined by positive blood testing or high suspicion of use by treating physicians based on history and clinical presentation. A dichotomous variable named minority status was created to encompass the following racial and ethnic groups: Black, Asian, Other which includes multiracial identification and Hispanic. Patients who identify as White race and do not identify as Hispanic based on ethnicity were excluded from this variable. Hospital disposition was defined as discharge to home, transfer for inpatient services, or death. In-hospital complications were treated as a composite score of complication burden based on the sum of the number of complications for each patient. The complications assessed were cardiac arrest, unplanned return to the operating room, unplanned intubation, unplanned extubation, inpatient alcohol withdrawal, ventilator-related pneumonia, and acute respiratory distress syndrome. 

Patient characteristics, including demographic and clinical variables and disposition, were compared across subgroups and defined based on the presence of unintentional TBI due to low-energy or high-energy falls. Chi-squared or Fisher’s exact test was used for categorical variables, and t-test or Wilcoxon rank sum test was used for continuous variables. A two-tailed a <0.05 was considered statistically significant. Logistic regression analyses were performed using fall energy (low or high separately) as the dependent variable. The following factors were examined as explanatory variables: sex, minority status, age, alcohol use, and insurance type. Multicollinearity was analyzed by calculating the variance inflation factor (VIF). We found no evidence of an impact of potential collinearity on the results of our model. The model calibration was assessed using the Hosmer-Lemeshow statistic and the discrimination by reporting the area under the receiver operating characteristic curve (AUC-ROC). Analyses were performed using SAS OnDemand for Academics (SAS Institute Inc., Cary, NC, USA). 

## Results

During the study period, 853 patients were evaluated by the hospital trauma service for unintentional falls with associated TBI. Table 1 describes the demographic and injury-related characteristics of the cohort. The average age of the TBI cohort was 71.5 years, and male sex accounted for approximately 59% (n=505) of the patients. Thirty-eight percent (n=324) of patients were classified as Black or Hispanic patients, and at least 20.1% (n=171) were of low socioeconomic status or had limited resources based on Medicaid insurance coverage use. Of the 853 TBI patients in the cohort, the percentages for mild, moderate, and severe TBI were 87.8% (n=749), 4.3% (n=37), and 7.9% (n=67), respectively. Concomitant fractures in the setting of unintentional falls were found in 15.6% (n=133) of patients. The average length of hospital stay for the cohort was 7.9 days; 10.5% (n=90) had an in-hospital complication, and 7.3% (n=62) of patients died during their initial hospitalization.

**Table 1:** Demographic and injury characteristics for the traumatic brain injury cohort (n=853) The data have been represented as N (sample size),(%), and Mean±SD. SD: standard deviation

Variable	Mean ± SD or n (%)
Age	71.5 ± 17.5
Sex	
Male	505 (59%)
Female	348 (41%)
Race/Ethnicity	
White	197 (23.0%)
Black	124 (14.5%)
Hispanic	200 (23.5%)
Asian	28 (3.3%)
Other	294 (34.5%)
Unknown	10 (1.2%)
Insurance	
Commercial	209 (24.5%)
Medicare	361 (42.3%)
Medicaid	171 (20.1%)
Other	61 (7.1%)
Unknown	51 (6.0%)
Glasgow Coma Scale score	13.9 ± 2.7
Traumatic Brain Injury Severity	
Mild	749 (87.8%)
Moderate	37 (4.3%)
Severe	67 (7.9%)
Injury Severity Score	13.8 ± 7.5
Orthopedic injuries	133 (15.6%)
Complications	90 (10.5%)
In-hospital death	62 (7.3%)
Total length of stay	7.9 ± 15.7

Of the 853 patients with unintentional falls and subsequent TBI in the cohort, low-energy and high-energy falls accounted for 760 (89.1%) and 93 (10.9%) patients, respectively. Table 2 describes the comparison of low- and high-energy fall patient characteristics. Patients with low-energy falls were older (72.8 vs 61.4 years​​; p<.0001) and had a greater proportion of Medicare usage (44.6% (n=339) vs 23.7% (n=22); p=0.0001). Low-energy fall patients also had a lower proportion of alcohol usage at the time of injury (14.0% (n=106) vs. 34.4% (n=32); p<.0001) and had a lower proportion of orthopedic injuries (13.8% (n=105) vs 30.1% (n=28); p<.0001) and other insurance usage (5.9% (n=45) vs 17.2% (n=16); p<0.0001). No significant differences between the two groups were found for sex, length of stay, ISS, GCS score on arrival, ICU and ventilator days, complication burden, commercial and Medicaid insurance usage, TBI severity, and disposition, including in-hospital death.

**Table 2:** Sample characteristics for low energy versus high energy unintentional TBI patients (n=853) The data have been represented as N (sample size), (%), Mean±SD, and associated Test statistics. P<0.05 is considered significant. Chi-square or Wilcoxon rank sum tests were used to calculate significance based on data type (count versus continuous data). ISS: Injury Severity Score; GCS: Glasgow Coma Scale; ICU: intensive care unit; TBI: traumatic brain injury

Variable	Low-energy fall	High-energy fall	Test statistic	Significance (p)
Age	72.8+16.8	61.4+19.3	-5.5594	<0.0001
Male, n (%)	445 (58.6)	60 (64.6)	1.2200	0.2694
Length of stay (mean+ SD)	7.5+ 13.2	6.5+ 7.2	-0.5969	0.5507
ISS (mean+ SD)	13.7+ 7.4	14.5+ 7.8	1.1784	0.2390
GCS score on arrival (mean+ SD)	13.9+ 2.8	13.5+ 3.0	-1.7928	0.0734
ICU days (mean+ SD)	1.9+ 4.8	2.0 + 3.4	0.7713	0.4407
Ventilator days (mean+ SD)	1.0+ 4.0	1.1 + 2.9	1.6952	0.0904
Alcohol present, n (%)	106 (14.0)	32 (34.4)	26.2458	<0.0001
Orthopedic injury, n (%)	105 (13.8)	28 (30.1)	16.7108	<0.0001
Complication burden (mean+ SD)	0.2 (0.6)	0.1 (0.4)	0.0205	0.9836
Insurance type				
Commercial, n (%)	179 (23.6)	30 (32.3)	3.3946	0.0654
Medicare, n (%)	339 (44.6)	22 (23.7)	14.7219	0.0001
Medicaid, n (%)	153 (20.1)	18 (19.0)	0.0312	0.8598
Other, n (%)	45 (5.9)	16 (17.2)	15.8876	<0.0001
Unknown, n (%)	44 (5.8)	7 (7.5)	0.4449	0.5047
TBI severity				
Mild, n (%)	672 (88.4)	77 (82.8)	2.4492	0.1176
Moderate, n (%)	32 (4.2)	5 (5.4)	0.2714	0.5878
Severe, n (%)	56 (7.4)	11 (11.8)	2.2768	0.1313
Disposition				
Home, n (%)	461 (60.6)	64 (68.8)	2.3309	0.1268
Inpatient facility, n (%)	243 (32.0)	23 (24.7)	2.0254	0.1547
Death during hospitalization, n (%)	56 (7.4)	6 (6.5)	0.1033	0.7479

Examination of bivariate relationships between fall height (continuous variable) and patient characteristics produced significant results as described in Table 3. Fall height was significantly associated with age. In high-energy fall patients, increasing fall height was associated with younger age. In low-energy fall cases, increasing fall height was associated with older age. Fall height also demonstrated a significant relationship with GCS score on arrival, ISS, and the number of ventilator days for high-energy falls. Increasing fall height was associated with lower GCS scores on arrival and greater ISS and ventilator days in patients with high-energy falls.

**Table 3:** Bivariate analysis identifying factors associated with fall height (n=853) The data have been represented as the estimated coefficient, associated 95% confidence interval, and test statistic. P<0.05 is considered significant. Bivariate linear regression analysis was used to determine significance. ISS: Injury Severity Score; GCS: Glasgow Coma Scale; ICU: intensive care unit; LE: low-energy; HE: high-energy

Variable	Univariate models B (95% CI)	Test statistic (t)	Significance (p)
Age (all patients)	-0.05 (-0.07 to -0.03)	-5.94	<0.0001
Age (LE fall patients)	0.04 (0.03 to 0.05)	2.55	0.0110
Age (HE fall patients)	-0.10 (-0.18 to -0.02)	-2.51	0.0140
GCS score on arrival (all patients)	-0.06 (-0.10 to -0.02)	-2.46	0.0141
GCS score on arrival (LE fall patients)	0.09 (-0.21 to 0.39)	0.61	0.5389
GCS score on arrival (HE fall patients)	-0.09 (-0.17 to -0.01)	-2.20	0.0303
ISS (all patients)	0.20 (0.08 to 0.32)	3.12	0.0019
ISS (LE fall patients)	0.18 (-0.60 to 0.96)	0.47	0.6385
ISS (HE fall patients)	0.36 (0.16 to 0.56)	3.63	0.0005
Length of stay (all patients)	-0.06 (-0.28 to 0.16)	-0.57	0.5714
Length of stay (LE fall patients)	-0.67 (-2.07 to 0.73)	-0.97	0.3323
Length of stay (HE fall patients)	0.04 (-0.16 to 0.24)	0.42	0.6774
ICU days (all patients)	0.03 (-0.05 to 0.11)	0.79	0.4324
ICU days (LE fall patients)	0.16 (-0.34 to 0.66)	0.64	0.5237
ICU days (HE fall patients)	0.06 (-0.04 to 0.16)	1.23	0.2224
Ventilator days (all patients)	0.04 (-0.04 to 0.12)	1.18	0.2366
Ventilator days (LE fall patients)	0.23 (-0.19 to 0.65)	1.09	0.2760
Ventilator days (HE fall patients)	0.09 (0.01 to 0.17)	2.01	0.0476

Regression modeling for the outcome of low-energy fall or high-energy fall with subsequent TBI produced similar results. The age of the patient and alcohol use predicted both fall types. Increasing age and reduced alcohol use were associated with low energy falls. Inversely, decreasing age and increased alcohol use were associated with high-energy falls. These data are depicted in Tables 4-5. Minority status, male sex, and insurance type variables did not produce statistically significant associations with fall energy type. These models showed moderate discrimination with an AUC of 0.7141 and good overall calibration.

**Table 4:** Low-energy fall odds estimates for TBI cohort variables The data have been represented as the point estimate and the associated 95% confidence interval. P<0.05 is considered significant. Multivariate logistic regression analysis was used to predict significant relationships between the variables of interest. TBI: traumatic brain injury; ChiSq: chi-square

Effect	Point estimate	95% confidence limits	Pr > ChiSq
Minority status	1.043	(0.641-1.697)	0.8663
Male sex	1.043	(0.627-1.735)	0.8723
Insurance (Medicaid)	1.675	(0.872-3.215)	0.1214
Insurance (Medicare)	1.839	(0.974-3.470)	0.0602
Insurance (Other)	0.495	(0.232-1.057)	0.0692
Age	1.019	(1.004-1.035)	0.0121
Alcohol use	0.451	(0.257-0.791)	0.0054

**Table 5:** High-energy fall odds estimates for TBI cohort variables The data have been represented as the point estimate and the associated 95% confidence interval. P<0.05 is considered significant. Multivariate logistic regression analysis was used to predict significant relationships between the variables of interest. TBI: traumatic brain injury; ChiSq: chi-square

Effect	Point estimate	95% confidence limits	Pr > ChiSq
Minority status	0.959	(0.589-1.561)	0.8663
Male sex	0.959	(0.576-1.596)	0.8723
Insurance (Medicaid)	0.597	(0.311-1.147)	0.1214
Insurance (Medicare)	0.544	(0.288-1.026)	0.0602
Insurance (Other)	2.020	(0.946-4.312)	0.0692
Age	0.981	(0.966-0.996)	0.0121
Alcohol use	2.217	(1.265-3.886)	0.0054

## Discussion

This study highlights the epidemiological and clinical differences in traumatic brain injuries resulting from low-energy and high-energy falls in an urban U.S. population, addressing critical gaps in the current literature. Our findings underline the importance of understanding these distinct mechanisms to effectively tailor prevention and management strategies.

Consistent with prior research, low-energy falls were predominantly observed among older adults, aligning with this population's increased frailty, diminished balance, and chronic comorbidities [6,8,9]. The greater prevalence of Medicare use among low-energy fall patients further supports the association of low-energy mechanisms with the geriatric population. Each year, approximately three million visits to U.S. emergency departments are due to falls in older adults [18], and geriatric falls cost Medicare approximately 29 billion dollars annually [2]. Several medical societies and organizations around the world have created fall prevention guidelines and recommendations based on available evidence and consensus by experts in fields such as geriatric and rehabilitation medicine [19]. These recommendations include medication review, environmental modifications, and exercise interventions, among others [19]. Strict implementation and adherence to fall prevention recommendations for older adults may help reduce future falls, TBI, and the poor outcomes associated with these injuries in this vulnerable population.

High-energy falls, in contrast with low-energy falls, were more common among younger individuals, likely reflecting occupational hazards, recreational risks, and higher rates of alcohol consumption. Alcohol use, in particular, emerged as a significant predictor of high-energy falls, underscoring its critical role as a modifiable risk factor for fall-related TBIs. These findings are consistent with prior studies emphasizing alcohol's contribution to fall risk across various populations [8,10]. Alcohol use is closely linked to TBI due to falls or other mechanisms of injury. It is estimated that between 30% and 50% of patients treated for TBI were intoxicated at the time of injury. These estimates increase when motor vehicle accidents and assault are the cause of injury [20]. In addition, the percentages of all TBI patients and TBI patients admitted for rehabilitation that meet diagnostic criteria for alcohol use disorder are up to 50% and exceed 50%, respectively [20, 21]. Previous studies have shown some success with brief ED interventions to reduce alcohol consumption [22]. Alcohol use reduction strategies, including patient education and resource linkage, may help reduce alcohol use, falls, and associated subsequent TBI.

Despite the significant differences in patient demographics, our study did not observe statistically significant differences in TBI severity, complication rates, or hospital mortality between low-energy falls and high-energy falls. This aligns with evidence suggesting that even low-energy mechanisms can lead to severe injuries, particularly in older adults, due to age-related physiological changes such as reduced bone density and impaired reflexes [9]. However, in high-energy fall patients, increasing fall height was associated with lower GCS scores and greater ISS, suggesting that energy transfer correlates with the extent of injury in this group. This result is consistent with the findings of Hallowell et al. demonstrating higher energy translates to greater injury severity [23]. However, in the literature, there has been some contention regarding the association between fall height and injury severity. There has been significant variability in the results, with some studies finding that fall height is a poor predictor of injury [24], while other studies find inconsistency in the relationship between these two variables [25,26]. The impacting body part was thought to affect the severity of injury after a fall, and this can vary based on the circumstances surrounding the fall, making it challenging to predict injury severity from fall height alone [24, 27]. Multi-regional trauma with injuries to the head and chest has been found to be associated with more severe injury and increased mortality [27].

Interestingly, in a bivariate analysis of fall height’s association with other study variables, low-energy fall patients demonstrated a higher average age compared to high-energy fall patients, which may reflect increased susceptibility to falls due to frailty and comorbid conditions [11]. The inverse relationship between age and fall height in low-energy fall patients suggests that younger individuals are more likely to experience falls from greater heights, potentially due to workplace exposures or risk-taking behaviors. Length of total hospital stay and length of ICU stay showed no association with fall height. This lack of association may be due to the presence of a significant percentage of older adults who are present in the low-energy fall group. Available data demonstrate that, on average, older adult patients have longer lengths of stay for hospital admissions than younger patients, who predominantly make up the high-energy fall group. This can be due to the medical complexities of care for older adults, their functional abilities, and the potential need for additional support that has to be thoughtfully planned among multiple care providers ahead of discharge [28]. One time-based variable, in particular, ventilator days, did show a significant positive association with fall height in patients with high-energy falls. Higher fall heights were associated with increasing ventilator days, an association likely attributable to injury severity increasing with fall height and the need for potential mechanical ventilation either due to more severe head injury or associated surgical procedures after injury. Further evaluation of this data is limited by the lack of variables for surgical procedures in this study’s trauma registry.

The findings of this study underscore the need and opportunity for targeted prevention strategies addressing both low-energy falls and high-energy falls. Fall prevention programs for older adults should focus on balance training, home modifications, and comprehensive medication reviews to mitigate fall risks. Additionally, public health campaigns targeting alcohol use and promoting safety measures in workplaces and recreational activities may help reduce high-energy falls in younger populations. We found a significant association between alcohol use and high-energy falls. Integrating routine alcohol screening and counseling into trauma care pathways could further support prevention efforts. Given the high proportion of individuals with potential TBI who present to emergency departments for evaluation, EDs may be an ideal location to screen for and educate trauma patients about alcohol use disorder.

Limitations and future directions

This study has several limitations that should be acknowledged. First, the single-center, retrospective design may limit the generalizability of our findings to broader populations. Second, our reliance on trauma registry data introduces potential biases, such as incomplete documentation of alcohol use or other confounding factors. Third, the lack of environmental and situational data precludes a deeper understanding of contextual factors contributing to falls. Fourth, due to a small sample size for this cohort, we may not be powered to detect the presence of some significant relationships between our variables of interest. 

Future research should prioritize multicenter, prospective studies to validate these findings across diverse populations. Additionally, qualitative investigations into patient-reported factors and environmental triggers may provide valuable insights into fall prevention strategies. Exploring the role of socioeconomic and cultural determinants could further inform tailored interventions for at-risk populations.

## Conclusions

Overall, patients with high-energy falls with associated TBI were younger and were more likely to develop fractures and utilize alcohol at the time of injury. High-energy falls did not lead to more severe injuries as demonstrated by nonsignificant differences in ISS, GCS, TBI severity, and death. Falls are a complex clinical issue due to the multitude of factors associated with increased risk. There is a need for context-specific research into the characteristics associated with falls that lead to subsequent TBI. A better understanding of this phenomenon will help to improve overall outcomes for fall patients, especially high-risk groups such as older adults.
